# Biocompatibility and Safety of 3D Printing Resins for Orthodontic Aligners: A Critical Review of Current Evidence

**DOI:** 10.3390/polym17223060

**Published:** 2025-11-19

**Authors:** Cecilia Goracci, Utkarsh Mangal, Stevan M. Čokić, Annalisa Mazzoni, Alessandro Vichi, Uros Josic

**Affiliations:** 1 Department of Medical Biotechnologies, University of Siena, 53100 Siena, Italy; cecilia.goracci@unisi.it; 2 Department of Oral Biology, Institute of Craniofacial Deformity, Yonsei University College of Dentistry, Seoul 03722, Republic of Korea; utkmangal@yuhs.ac; 3 Department of Oral Health Sciences, BIOMAT-Biomaterials Research Group & UZ Leuven, Dentistry, KU Leuven, B-3000 Leuven, Belgium; stevan.cokic@kuleuven.be; 4 Department of Biomedical and Neuromotor Sciences (DIBINEM), University of Bologna, 40125 Bologna, Italy; annalisa.mazzoni@unibo.it; 5 Dental Academy, University of Portsmouth, Portsmouth PO1 2QG, UK; alessandro.vichi@port.ac.uk

**Keywords:** 3D printing, clear aligners, biocompatibility, biofilm, orthodontics

## Abstract

Orthodontic aligners 3D-printed in resin currently provide a viable alternative to thermoformed ones. However, concerns have been raised regarding their biocompatibility. This review addressed the available scientific evidence on the biological properties of marketed resins for 3D printing of orthodontic aligners, encompassing cytotoxicity, estrogenicity, biofilm formation, and oral soft tissues reactions. A comprehensive literature search of several databases was conducted and PRISMA guidelines were followed to summarize the retrieval. Eleven studies were included in the review. They provided information on only three marketed resins: Tera Harz TC-85 DAC and Tera Harz TA-28 (Graphy) and Clear-A (Senertek). For the last two materials, only one investigation has been performed. Despite the large variability in experimental protocols, the lack of cytotoxicity of Tera Harz TC-85 DAC was a consistent finding. Also, no estrogenic effect was detected for this resin, in line with the lack of any bis-phenol A precursor in its chemical composition. In two clinical studies, oral soft tissue reactions were reported as rare and non-serious occurrences. Biofilm adhesion was regarded as critical for the clinical safety of 3D-printed aligners. Standardization of in vitro protocols, also including more clinically relevant settings, chemical characterization of the resins’ eluates, and collection of additional in vivo data are advised to improve the quality of the evidence.

## 1. Introduction

The evolution of 3D printing in Dentistry has enabled the in-office production of several dental products. Among them interest has particularly grown for 3D-printed aligners, as a reflection of the increasing demand for esthetic orthodontic treatment. These aligners are fabricated through a layer-by-layer process in which photopolymerizable resins, such as the ones based on methacrylate or urethane dimethacrylate formulations, undergo polymerization and form a cross-linked network upon exposure to specific wavelengths of light during printing [1,2,3,4]. Also named as directly printed aligners (DPAs), these appliances hold the promise to overcome some of the limitations of thermoformed devices, and their favorable characteristics have been elucidated in several recent summaries of evidence [5,6,7]. Advantages have been identified in the effectiveness of the workflow, eliminating the need for model printing, aligner trimming and polishing, as well as in the accuracy of the printed devices that exhibit uniform thickness, unless otherwise designed, and achieve better adaptation to undercut areas. The improved fit also translates into augmented grip that may reduce the need for attachments. The extended design options are additional benefits of directly printed aligners [8,9,10]. The adjunct of pressure points or columns by locally changing the thickness of the print and the modeling of bite ramps, posterior bite blocks, and advancement wings allow for better control of tooth movement. Still, the major advancement in the biomechanical behavior of DPAs is allegedly provided by the shape memory ability of the 3D printable resins. Shape memory eases initial seating of the aligner, while reduced stress relaxation has been claimed to diminish the number of aligners needed per movement [11,12,13].

Shape memory ability has been declared for some marketed resins: Tera Harz TC-85 DAC and Tera Harz TA-28 (Graphy, Seoul, Republic of Korea); OD-Clear TF LTP (3Dresyns, Barcelona, Spain); Luxcreo (Luxmark, Belmont, MA, USA); and Clear-A (Senertek, Ismir, Turkey). Among them, only Tera Harz TC-85 DAC (TC-85) has been suitably investigated with in vitro and in vivo studies, addressing several clinically relevant properties, such as mechanical behavior, fit, chemical stability, and optical characteristics [14,15,16,17,18,19,20,21,22,23,24,25,26,27,28,29,30,31,32,33,34]. Among the clinically relevant characteristics of DPAs, biocompatibility is of particular importance [35,36,37,38,39,40,41,42,43,44,45]. Considering that aligners are worn for approximately 22 h per day over several months, these materials are in prolonged and continuous contact with intraoral tissues and fluids [42]. This constant exposure raises concerns about the potential release of residual monomers or degradation products into the oral environment. Moreover, because new aligners are introduced every 7–14 days, any initially elevated elution from DPAs may cumulatively result in sustained exposure throughout treatment. Therefore, the potential biological risks associated with such repeated contact warrant thorough investigation [43]. Although Dantagnan et al. [42] systematically addressed the literature on biocompatibility of DPAs, this review focused exclusively on in vitro evidence, highlighting the need for a more comprehensive evaluation that integrates available clinical data.

Therefore, the present review was aimed at comprehensively and critically revising the in vitro and in vivo evidence so far collected on the biologic properties of DPAs, encompassing cytotoxicity, estrogenicity, allergic potential, and biofilm adherence, with the ultimate goal of providing guidelines for a safe clinical use of DPAs.

## 2. Materials and Methods

### 2.1. Search Strategy

This review was conducted in adherence to the Preferred Reporting Items for Systematic Reviews and Meta-Analysis (PRISMA) statement [46]. An electronic search with no language or date restrictions was conducted in Medline via PubMed, Scopus, Web of Sciences, and Cochrane library databases. The following keywords were used: “3D printed aligner” OR “3-D printed aligner” OR “direct printed aligner” AND “biocompatibility” OR “cytotoxicity” OR “estrogenicity” OR “genotoxicity” OR “allergy” OR “hypersensitivity” OR “biofilm”, adapted to the specific database (Table 1). The last search update was performed on 20 July 2025.

Further manual searching was carried out by screening the list of references of the initially retrieved articles and exploring the websites of the relevant journals.

### 2.2. Eligibility Criteria

It was established to include in the review any original research that, regardless of the design, assessed whether resins for 3D printing of orthodontic aligners met the requirements for safe intraoral use. The exclusion criteria were set so as to leave out conference abstracts, editorials, and review papers.

### 2.3. Study Selection, Data Collection, and Data Items

A two-stage selection process was performed independently by two authors (C.G. and U.J.), who first screened the articles by title, keywords, and abstract, then read the full texts and eliminated the papers that did not comply with the inclusion criteria. Using custom-made extraction forms in MS Word, the two reviewers extracted the following data: authors and year of publication, resin chemical composition, manufacturing method, type of biocompatibility test (extraction solvent, incubation period, cell line, exposure of cell to potential toxic), type of microbial adhesion test, tested microorganisms, adherence of test protocols to international standards, time-points, results, and conclusions.

## 3. Results

**Figure 1:**
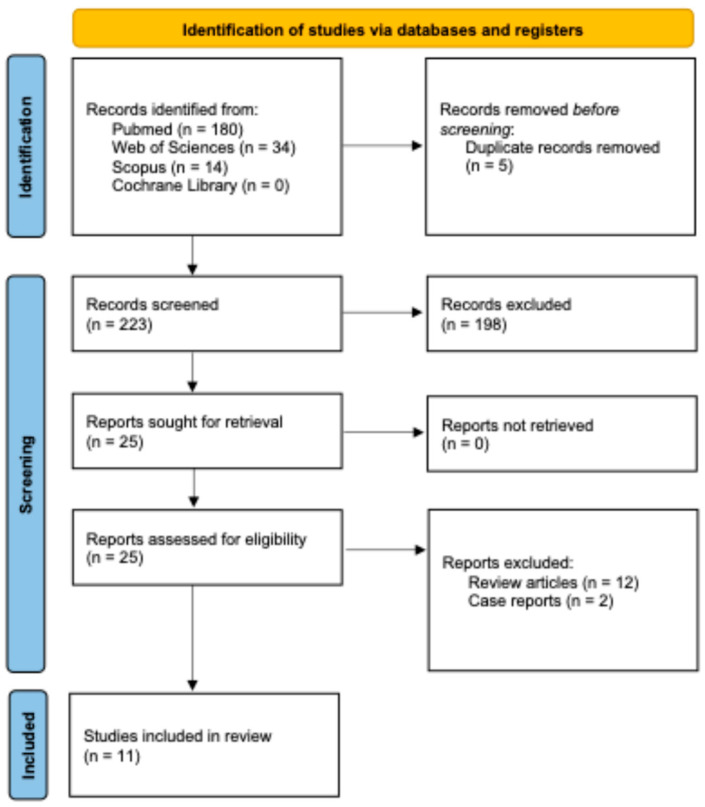
Flowchart according to the PRISMA statement.

Figure 1 illustrates the study selection process. After removal of duplicates and applying the exclusion criteria, 11 articles (Supplementary Table S1) were included in this review.

### 3.1. In Vitro Tests of Leaching, Cytotoxicity, and Estrogenicity

Three marketed resins for 3D printing of orthodontic aligners have been tested for biocompatibility: TC-85 [15,23,25,35,36,39], Tera Harz TA-28 (TA-28) [14], and Clear-A (C-A) [15]. The chemical compounds of the TC-85 resin as disclosed by the manufacturer are aliphatic vinyl ester-urethane monomers, with acrylate and/or methacrylate functionalization [11]. Recently Choi et al. [10] reported that the resin is formulated from GR30860 and GR3060 oligomers, and features 11 bis(2,4,6-trimethylbenzoyl)-phenylphosphine oxide, commercially known as Irgacure 819 (BASF SE, Ludwigshafen, Germany), as the initiator of light-polymerization. No information is currently available regarding the chemical composition of TA-28 and C-A resin.

In the study by Willi et al. [35], aligners printed with TC-85 resin were centrifuged for 4 min and post-polymerized with the Cure M curing machine (Graphy, Seoul, Republic of Korea) for 24 min on the inner and the outer sides. Aligners were stored in distilled water at 37 °C for 1 week and eluates were analyzed by liquid chromatography/mass spectrometry for the determination of urethane dimethacrylate (UDMA) and bispenol-A (BPA). UDMA release was detected for all the specimens and ranged between 29 and 96 μg/L. No BPA elution was recorded from any of the specimens. The authors concluded that the variability in UDMA elution may be a concern for potential health hazard. It should however be noted that the degree of monomer conversion measured in the study was 83%, a percentage lower that that recorded in later studies, where a curing machine equipped with a nitrogen chamber, Tera Harz Cure THC2 (Graphy, Seoul, Republic of Korea), was utilized to provide an oxygen-deprived post-polymerization environment [26,31].

TC-85 aligners manufactured with the same protocol as in the study by Willi et al. were tested for cytotoxicity and estrogenicity by Pratsinis et al. [39]. Aligner pieces were incubated in sterile deionized water at 37 °C for 14 days. The cytotoxicity of eluates was assessed by means of MTT (3-[4,5-dimethylthiazol-2-yl]-2,5-diphenyltetrazolium bromide) assays on human gingival fibroblasts (HGFs). The oxidative stress of the eluates was also determined by measuring the HGF intracellular reactive oxygen species (ROS) levels. In addition, the estrogenicity of aligner material eluents was tested by measuring their proliferative effect on estrogen-sensitive human breast adenocarcinoma cells (MCF-7s) with the E-screen assay method. The authors found that the factors released by TC-85 aligners were not cytotoxic for HGFs and did not place these cells under oxidative stress. Also, no estrogenic effect was reported.

Campobasso et al. investigated the influence of the post-polymerization procedure on cytotoxicity by comparing TC-85 aligners cured in the presence or absence of oxygen, respectively, with Tera Harz Cure THC2 and FormCure (Formlabs, Somerville, MA, USA) devices [36]. Aligner specimens were placed in Dulbecco’s Modified Eagle Medium (DMEM), where mouse calvaria pre-osteoblasts (MC3T3E-1) had been cultured. The incubation period was 14 days and cell viability was measured with the MTT assay. Nitrogen-rich post-polymerization yielded highly biocompatible products, while specimens cured in the presence of oxygen exhibited moderate cytotoxicity.

Still concerned with post-curing, Iodice et al. [25] focused on the effect of polymerization time on cytotoxicity of TC-85. Resin samples were incubated in saliva for 14 days, then the supernatant was collected to expose HGFs to the possibly released factors. HGFs were also plated on resin samples, as well as on a glass sample that served as control. Cell viability was assessed with the MTT assay and compared to that of HGFs exposed to samples of PET-G thermoformed aligner material. For TC-85 a cytotoxic effect similar to that of the thermoformed material was observed. An inverse relationship emerged between curing time and cell viability, and 50 min curing resulted in a significant reduction in HGF growth. Based on these findings, the authors recommended strict adherence to the 14 min post-curing time indicated by the resin manufacturer.

Additional variables in the post-processing possibly influencing biocompatibility by affecting the elimination of residual uncured monomers are centrifugation time and temperature, and they have been addressed in the study by Kim et al. [23]. The authors mentioned that the cytotoxicity test protocol adhered to ISO 10993-5. Aligner specimens were sterilized with EO gas and incubated in RPMI 1640 culture medium for 24 h. The tested cell line was L-929 (mouse fibroblasts), and cell viability was evaluated with MTT assays. TC-85 aligners cleaned with centrifugation for 2, 4, or 6 min at room or high (55 ± 2 °C) temperature, as well as with immersion in isopropyl alcohol (IPA) were all found to be biocompatible according to ISO 10993-5 standards.

These same standards were met in the study by Bleilöb et al. [14] by specimens of TA-28, a resin for DPAs recently introduced on the market. Aligner pieces were incubated in culture medium or saliva for 12 days, simulating the wear duration of each aligner in the clinical setting. The medium or saliva was then transferred to a well plate containing HGFs. Cell viability was measured with Alamar Blue assay. The resin, polymerized with the proprietary curing device for the recommended time of 20 min, proved to be biocompatible even with a thickness of 6 mm, simulating areas of the aligner designed to produce specific tooth movements or auxiliaries. Concurrently, exposure to saliva was found to affect cell viability.

Bor et al. [15] tested the cytotoxicity of TC-85 and C-A rectangular specimens sized 10 × 12 × 0.6 mm and printed with a layer thickness of 100 µm. Post-polymerization was performed for either resin with the Tera Harz Cure THC2 machine. For C-A an additional group of specimens was post-cured within a glycerol-filled glass device named Curie Cure (Ackuretta, Taipei City, Taiwan). The ISO 10993-12 standard was followed in the preparation of the extracts of the printed specimens. After sterilization by immersion in 70% ethanol for 5 min, specimens were placed in DMEM at 37 °C for 72 h. HGFs and XTT assay were utilized for the assessment of cell viability. Additionally, a technique for real-time cell analysis (xCEELLigence DP, Agilent, Santa Clara, CA, USA) was employed to measure cell doubling times. Undiluted extracts had limited cytotoxicity at 24 h, though cell viability remained above the 70% threshold established by ISO 10993-5. At 48 and 72 h an increase in cellular viability was recorded, indicating the potential of the cells to adapt and recover the proliferation ability.

### 3.2. Clinical Studies

Data on clinical safety of 3D-printed aligners were provided by two clinical studies [29,40].

Migliorati et al. [29] published the outcome of a prospective pilot study on 17 patients treated with TC-85 resin aligners (0.5 mm thickness, printed in successive 100 µm thick layers). The occurrence of lip swelling was reported by one patient while wearing the 4th aligner. The reaction was ascribed to inadequate curing of the aligner material. Treatment was prosecuted with the following aligner in the series and completed without any other complication. Knode et al. observed 54 patients treated with TC-85 aligners over a 12-month period [40]. Moderate burning sensations were referenced by three participants in the study. Two of them also experienced minor tissue reactions.

### 3.3. Evaluation of Biofilm Formation

Wu et al. treated the surface of TC-85 aligners with a blend of 2-hydroxyethyl methacrylate (HEMA) and carboxybetaine methacrylate (CBMA) solution at different stages of their fabrication (before post-curing, after post-curing, and after post-processing) [44]. The authors then evaluated the effect of surface treatment on physico-mechanical characteristics, as well as on bacterial biofilm formation. Using confocal laser microscopy, the formation of salivary biofilm on disc-shaped resin specimens was observed over a period of 72 h. The crystal violet assay was employed to quantify the adhesion of *Streptococcus mutans*. Biofilm formation and *Streptococcus mutans* adhesion were significantly reduced when surface treatment was performed after post-curing. The procedure did not significantly affect the physico-mechanical properties of the aligner material.

Pasaoglu Bozkurt et al. used *Streptococcus mutans* and *Lactobacillus acidophilus* standard strains, violet staining method, and spectroscopy to assess the amount of biofilm formation over time on TC-85 aligners in comparison with thermoformed aligners [45]. They reported that at 7 days significantly more biofilm formation was observed on TC-85 than on Smartee or Invisalign. Given that bacterial adhesion increases with higher intrinsic surface roughness, the inherent microroughness imparted by 3D printing of UDMA-based matrices is considered a potential contributor to the increased biofilm accumulation observed on TC-85 aligners. Although microtopographic irregularities have been associated with the intraoral wear of PETG-based aligners, the micro-irregularities inherent to the additive 3D printing process are also believed to further facilitate microbial colonization. These findings highlight the importance of optimizing printing parameters and nitrogen-assisted curing protocols to reduce surface heterogeneity and biofouling potential.

## 4. Discussion

When assessing the evidence available on the biologic properties of materials for DPAs, the first striking finding was that limited information has only been provided for TC-85 regarding the chemical compounds of the resin. For all the other marketed resins, the manufacturers have so far kept the chemical composition undisclosed under the claim of patent protection. However, in this regard, Turkalj et al. [47] recently commented that since REACH and Medical Device Regulations require that ingredients of medical devices be revealed [48,49], it should be verified to what extent manufacturers of DPA resins are complying with these rules. Turkalj et al. also reported that clinicians and researchers often face difficulties in retrieving clear and complete information about the chemistry of medical devices and advocated greater transparency from the manufacturers [47].

Another relevant observation emerging from this review was that the experimental setups of in vitro cytotoxicity tests varied largely, thus precluding a quantitative combination of the studies’ results.

Not only for comparative purposes, but also for a safe clinical use, adherence to manufacturer’s instructions has been recommended in the handling of 3D-printed resins. A particularly critical step for its bearing on biocompatibility is represented by post-polymerization. For the Graphy resins the indication is that post-curing should be performed in an oxygen-free atmosphere, provided by a nitrogen generator [13,15,23,25,29,36,40]. However, the earlier studies on TC-85 resin by Willi et al. [35] and Pratsinis et al. [39] did not comply with this requirement. The less-than-ideal monomer to polymer conversion achieved in the presence of oxygen may have accounted for the wide-ranging elution of UDMA monomers reported in the study by Willi et al. [35]. Campobasso et al. validated the beneficial effect for biocompatibility of post-curing the resin in an oxygen-depleted environment [36].

In three studies aligners were sterilized prior to use in the experiment in different ways: by means of steam at 121 °C temperature [36], ethylene oxide (EO) gas [23], or 70% ethanol solution [15]. These various procedures may differently affect the physical properties and chemical stability of the resins. According to Bor et al., immersion in ethanol is preferable as EO residues may affect cytotoxicity per se [15].

In all the published studies on cytotoxicity of 3D-printed aligner extracts, tests involving the exposure of the cells to eluates of the materials were performed. This in vitro model is more commonly utilized in dental research than direct contact tests, where the materials are in direct contact with the cells, or indirect contact tests, where materials and cells are separated by a barrier e.g., an agar layer or a filter. Only Iodice et al. performed a 3-day contact test between aligner specimens and HGFs, in addition to the eluate exposure [25].

However, among extract test protocols differences emerged in the choice of the medium used to extract eluents from aligner specimens. Double distilled water [35], sterile deionized water [39], saliva [25], and cell culture medium [13,15,23,36] were utilized in the different experiments. Human saliva can be considered as the most clinically relevant extraction solution [50], and pH changes, enzymatic activity, and microbial interactions occurring intraorally are relevant conditions for aligner elution and should ideally be reproduced for reliable biocompatibility testing. DMEM has demonstrated an extraction potential similar to saliva [51]. Furthermore, the volume of the medium can affect the degree of elution from the tested material. It is stated in ISO 10993-13:2010 that the ratio between sample surface area and volume of extraction solution should be at least 0.2 g/mL [38]. However, only Bor et al. [15] and Kim et al. [23] reported that such ratio was maintained in their study.

Variability was also seen in extraction times, as incubation periods of 24 h [23], 72 h [15], 7 days [35], 12 days [13], and 14 days [25,36,39] were reported. Twenty-four hours is the storage time indicated in the ISO 10993-5 when extraction is conducted at a 37 °C temperature. Longer incubation periods may lead to higher elution of compounds, potentially increasing observed cytotoxic effects [52]. However, longer extraction times, such as 12 or 14 days, were chosen as they represent the recommended wear time for each aligner. Moreover, Bor et al. observed that a prolonged incubation model would better elucidate long-term and cumulative biological effects of eluates [15].

A lack of standardization was also evident in the selection of the cell type. Human gingival fibroblasts (HGFs) [12,14,25,39], mouse calvaria pre-osteoblasts (MC3T3E-1) [36], and mouse fibroblasts (L-929) [23] were tested in the different studies. It has previously been demonstrated that different cell types exhibit different sensitivities towards dental materials. Specifically, human fibroblasts have been shown to be more sensitive than mouse fibroblasts, and human pulp fibroblasts more sensitive than human gingival fibroblasts. The use of HGFs is recommended by ISO 10993-5 [52] as they represent the cells most directly exposed to aligner eluates. However, Bor et al. [15] suggested that future studies should also include other relevant oral cell lines, such as oral keratinocytes and periodontal ligament fibroblasts, to better capture the tissue specific responses.

Apart from the choice of cell type, researchers increasingly emphasize that the testing model should more closely reproduce the in vivo tissue microenvironment. Conventional monolayer assays fail to capture the complex cell–cell and cell–matrix interactions that are essential for mucosal homeostasis [53]. Organotypic co-culture systems combining keratinocytes and fibroblasts can reproduce epithelial stratification and barrier function more accurately than single-layer cultures. Furthermore, the development of three-dimensional (3D) tissue-engineered oral mucosa models, such as EpiGingival^TM^, EpiOral^TM^, or self-assembled oral mucosa equivalents, has provided a more physiologically relevant platform for toxicological evaluation, allowing both histological and cytokine-based assessment of tissue responses and mucosal irritation. More recently, dynamic perfusion and microfluidic “oral-mucosa-on-a-chip” or “tooth-on-a-chip” systems have been introduced to simulate intraoral parameters such as pH, salivary flow, temperature, and mechanical stress, while enabling real-time quantification of inflammatory biomarkers including IL-1α, IL-1β, and TNF-α [53,54]. Integrating these advanced 3D and microphysiological models with standardized ISO-based cytotoxicity assays and quantitative analysis of released compounds would significantly enhance the reproducibility, physiological relevance, and clinical predictivity of in vitro biocompatibility testing for 3D-printed aligner resins.

Concerning with the method to measure cell viability, MTT assay is a method validated in ISO 10993-5 [52] and was utilized in all the investigations except the studies by Bleilöb et al. [14] and Bor et al. [15]. MTT assays assess cell viability by spectrophotometric quantification of formazan, a colored product formed when tetrazolium salts are reduced by mitochondrial enzymes in metabolically active cells. In contrast, Bleilöb et al. [14] employed the Alamar Blue assay, which measures overall cellular metabolic activity, rather than focusing solely on mitochondrial function. This assay relies on the reduction of resazurin, a non-fluorescent blue dye, to resorufin, a pink and fluorescent product, reflecting cellular redox activity and correlating with cell viability and metabolic health. XTT assay is also an ISO-certified method [52]. Bor et al. [15] described XTT assay as a well-established method largely utilized to test dental materials and real-time cell analysis as a more recent method to monitor cell dynamics, jointly providing a reliable assessment of cytotoxicity [15].

Currently, the prevailing understanding of monomer toxicity implicates oxidative stress pathways, with reactive oxygen species (ROS) production serving as an early cellular response to resin monomers. However, ROS generation was investigated only in the study by Pratsinis et al., who found it to be unaltered in HGFs exposed to eluates of Tera-Harz TC-85 aligners [39]. The study by Pratsinis et al. was also the only one assessing the estrogenicity of the resin. No estrogenic effect was detected, and the observation was in line with the finding of a lack of bis-phenol A (BPA) leaching from TC-85 resin reported by Willi et al. [35]. These authors described the resin as composed of aliphatic vinyl ester-urethane monomers, with acrylate and/or methacrylate functionalization, and noted a largely variable release of UDMA, warranting further investigation of the potential toxicity. It is however worth reiterating that a relatively low degree of monomer to polymer conversion was measured for the tested aligners, possibly in relation to the post-curing procedure being performed in an oxygen rather than a nitrogen atmosphere [35]. The release of UDMA monomers should therefore be measured in resin specimens subjected to post-curing under nitrogen-saturated conditions, which is currently recommended by the manufacturer and has yielded degree of conversion values exceeding 97% [26,31].

Bor et al. advocated the employment of more refined analytical techniques, such as gas chromatography–mass spectroscopy, in order to gain a quantification of specific monomer concentrations in extracts and therefore a better understanding of their potential toxicity [15]. The latter should also include the assessment of genotoxicity of 3D-printed resins for aligners. The concern of a potentially damaging effect on DNA was raised by Willi et al. for the UDMA eluates of TC-85 aligners [35]. However, the issue has not been properly addressed so far in the literature, while it is questionable whether the UDMA release recorded in the experiment by Willi et al. adequately reflects the clinical condition.

When considering whether the experimental protocol included testing a control material for comparative purposes with the 3D-printed resins, only the study by Iodice et al. had this feature and to serve as control was thermoformed PET-G [25].

As for adherence of the experimental protocols to testing standards, only Kim et al. [23] and Bor et al. [15] mentioned referring to ISO 10993-5, which applies to tests for in vitro cytotoxicity, as a part of the biological evaluation of medical devices [52]. Clearly, standardization of research protocols would make the comparisons across studies more meaningful and would more valuably contribute to the overall understanding of DPAs’ biocompatibility.

Despite the methodological inconsistencies among studies, the lack of cytotoxicity of TC-85 resin was a recurrent finding in all the reviewed papers. Also, C-A was reported by Bor et al. to be biocompatible with HGFs [15].

It should however be mentioned that, for a more clinically relevant assessment of eluate cytotoxicity, resin specimens should be subjected to cyclic mechanical loading, changes in pH and temperature, and bacterial and salivary enzymatic activities meant to simulate the intraoral conditions [15,42]. Currently available published cytotoxicity studies have indeed been criticized for being merely based on passive elution, thus overlooking the potential effect on biocompatibility of resin degradation with function [42].

Overall, the clinical evidence so far acquired on the biocompatibility of 3D printed aligners is scarce. Safety has been assessed in vivo only for the TC-85 resin and only in two studies [29,40] with relatively small sample sizes. Still, the emerging clinical picture was that adverse reactions were rare and non-serious, as the adherence to manufacturer’s instructions was strict [29,40]. Yet, further investigations would be desirable in order to strengthen the clinical evidence, possibly addressing autoimmune diseases, heightened sensitivity to resin components in artificial nail attachment [55], and gingival biotype as potentially influential conditions. Also, as the clinical use of DPAs has been increasing, it can also be expected that data from larger samples will be provided in the near future.

It may also be worthwhile to verify whether the indication to immerse acrylic resin products, such as removable dentures, in water for 24 h before their first intraoral use provided in previous prosthodontic literature [15,56,57,58] may be applicable also to DPAs with the objective to wash away uncured monomer remnants that may cause mucosal irritation. Bor et al. interpreted the decrease in cell viability recorded at 24 h as the effect of water-soluble residual monomers that rapidly leach from the resin in an aqueous environment [15]. The authors thereby endorsed rinsing or soaking the aligner in water before first use as a simple way to limit the initial exposure to leachates; thus, overall improving the device biocompatibility.

Biofilm formation and material degradation represent significant challenges in orthodontic treatment with clear aligners. With specific regard to TC-85 resin, it should be considered that, based on the earlier literature, the content of UDMA monomers may make the resin susceptible to biofouling [59,60]. Concerning the reviewed studies on biofilm adhesion to DPAs, it should be noted that short-term in vitro protocols may fail to capture the complexity of intraoral biofilm development as well as the effect of intraoral aging on surface characteristics of the material. Additionally, reliance on single or limited microbial strains may underestimate the diversity of oral flora. Furthermore, while innovative approaches like chitosan nanoparticles or carboxybetaine-based coatings show promise, their durability under functional loads and saliva flow remains uncertain.

Consistent with the findings of Pasaoglu Bozkurt et al. [45], the higher biofilm accumulation observed on TC-85 aligners compared to thermoformed counterparts likely reflects both material- and surface-related factors. Clinically, these findings underscore the importance of strict hygiene measures and reinforce the need for aligner hygiene protocols that suppress bacterial adhesion without compromising mechanical or optical performance. Optimized post-curing procedures and higher-resolution printing may also enhance biofilm resistance and long-term oral compatibility.

Across these studies, standardizing biofilm quantification metrics, expanding microbial panels, and conducting experiments under simulated or in vivo conditions would be crucial for validating findings and improving the reliability of the evidence. Also, with specific regard to the Bozkurt et al. study [45], it should be noted that no information was provided about the fabrication procedure of TC-85 specimens and only the detail that print layer thickness was 100 µm could be extrapolated from the text. Conversely, 50 µm printing has been advised for the purpose of reduced surface roughness.

Lastly, it is worth mentioning that no study has so far specifically addressed the genotoxicity of 3D-printed resins for aligners. Willi et al. [35] raised the concern of potential genotoxicity of UDMA eluates from TC-85 aligners. However, it should be noted that the protocol followed in this investigation has some limitations in its clinical relevance, as aligners were not cured according to the current manufacturer’s instruction.

## 5. Conclusions

The chemical composition of resins for 3D printing of orthodontic aligners has been disclosed to a limited extent only for TC-85.

Despite a large heterogeneity of the in vitro protocols, all the studies indicated that TC-85 resin was not cytotoxic for a variety of tested cell lines.

Also, no estrogenic activity was detected for TC-85 resin, in line with the absence of bis-GMA monomers in the chemical composition.

The clinical evidence on the biologic safety of DPAs currently consists of two studies with relatively small sample sizes. Lip swelling, mouth burning, and mucosal reactions were described as rare, non-serious occurrences, with a favorable prognosis, not affecting the prosecution of treatment. The cruciality of strictly following the manufacturer’s instructions in DPAs’ manufacturing was highlighted; soaking DPAs in water before their first use may improve the devices’ biocompatibility.

Future research should aim at reinforcing the evidence regarding biosafety of DPAs by conducting well-organized clinical trials with bigger sample sizes, as well as at enhancing the anti-biofilm properties and resistance to intraoral degradation of DPAs’ materials.

Also, effort should be made by clinicians and researchers to refine the manufacturing procedure of DPAs and to standardize the experimental settings by adhering to recently published guidance on the assessment of biocompatibility of biomaterials, as well as existing ISO standards. Finally, a greater transparency about the chemical composition of the materials is expected from the manufacturers’ side, with all of this leading to a more reliable prediction of DPAs’ biological safety.

## Figures and Tables

**Table 1:** Electronic databases, search strategies, and number of documents retrieved for each database.

Electronic Database (Number of Hits on 20 July 2025)	Search Strategy
**Medline via PubMed** (n = 180) https://pubmed.ncbi.nlm.nih.gov	((3d AND printed AND aligner) OR (3d- AND printed AND aligner) OR (direct AND printed AND aligner)) AND (biocompatibility OR cytotoxicity OR estrogenicity OR genotoxicity OR allergy OR hypersensitivity OR biofilm)
**Web of Science** (n = 34) https://www.webofscience.com	TS = ((“3D printed aligner” OR “clear aligner” OR “orthodontic aligner”) AND (material * OR resin * OR polymer *)) AND TS = (biocompatibility OR toxicity OR degradation OR biofilm)
**Scopus** (n = 14) https://www.scopus.com	(TITLE-ABS-KEY (“3D printed aligner”) OR TITLE-ABS-KEY (“3D-printed aligner”) OR TITLE-ABS-KEY (“direct printed aligner”) AND TITLE-ABS-KEY (biocompatibility OR cytotoxicity OR estrogenicity OR genotoxicity OR allergy OR hypersensitivity OR biofilm))
**Cochrane Library** (n = 0) https://www.cochranelibrary.com	(“3D printed aligner” OR “3D-printed aligner” OR “direct printed aligner”) AND (biocompatibility OR cytotoxicity OR estrogenicity OR genotoxicity OR allergy OR hypersensitivity OR biofilm)

## Data Availability

No new data were created or analyzed in this study. Data sharing is not applicable to this article.

## Supplementary Materials

The following supporting information can be downloaded at: https://www.mdpi.com/article/10.3390/polym17223060/s1, Table S1. Characteristics of the studies included in the review.
