# Supplementary material for: Biocompatibility and Safety of 3D Printing Resins for Orthodontic Aligners: A Critical Review of Current Evidence
Source: Polymers (Basel). 2025 Nov 19;17(22):3060. doi: 10.3390/polym17223060 (PMC12656306; doi:10.3390/polym17223060)
Supplement: Supplementary file 1 [file polymers-17-03060-s001.zip › polymers-3822599-supplementary.pdf]

Supplementary Table S1. Characteristics of the studies included in the review.

Study ID	Study design	Objective	Material tested	Manufacturing method (3D printer)	Sample size / shape	Sterilization / disinfection method	Cell line and test type	Extraction medium and incubation period	Results	Conclusions
Willi et al. (2022)	In vitro	To quantitatively assess the degree of conversion and the water-leaching targeted compound from 3D-printed aligners	Tera Harz TC85A aligner resin (Graphy, Seoul, South Korea)	Sprintray Pro 55 3D printer (Sprintray, Los Angeles, California, USA)	n=5 (degree of conversion); n=10 (monomer release)	N.I.	N.A.	Double distilled water, 7 day	The resin was composed of aliphatic vinyl ester-urethane monomers, with acrylate and/or methacrylate functionalization. The degree of conversion was estimated as to 83%. There was no detection of BPA in any of the assessed samples (0.25 μg/l). Quantifiable amounts of UDMA were detected in all the exposed samples, ranging from 29 to 96 μg/l	Although efficiently polymerized and BPA free, the great variability in the amount of UDMA monomer leached from the examined samples may raise concerns on potential health hazards after repeated intraoral exposure, which is indicated for this class of materials
Pratsinis et al. (2022)	In vitro	To investigates	Tera Harz TC85A	Sprintray Pro 55 3D printer	n=20 per group (in	N.I.	Human gingival fibroblast strain;	Sterile deonized	3 days for MTT test, overnight	If any factors were released

Study ID	Study design	Objective	Material tested	Manufacturing method (3D printer)	Sample size / shape	Sterilization / disinfection method	Cell line and test type	Extraction medium and incubation period	Results	Conclusions
		the cytotoxicity and estrogenicity of a 3D-printed orthodontic aligner by assessing its biological and behavioral effects	aligner resin (Graphy, Seoul, South Korea)	(Sprintray, Los Angeles, Calif)	total 60), each aligner was cut into 3 smaller pieces		Extract test (14-day elution), modified MTT test, ROS and E-screen for estrogenicity	water, 14 days	for ROS analysis, 6 days for E-screen test	during the 14-day aging of 3D-printed aligners in water, these were not found to be cytotoxic for human gingival fibroblasts and did not affect their intracellular reactive oxygen species levels. Moreover, no estrogenic effects of these putative eluates were observed based on an E-screen assay
Campobasso et al. (2023)	In vitro	To assess the in-vitro cytotoxicity of 3D-printed aligners using different post-	Tera Harz TC-85 DAC resin (Graphy, Seoul, South Korea)	3D-printer (AccuFab-L4D, Shining 3D Tech. Co., Ltd., Hangzhou, China)	n=12, aligners were cut into smaller pieces	121 ºC	Pre-osteoblast mouse calvaria MC3T3E-1 (CRL-2593, American type culture 257 collection,	DMEM, 7 and 14 days	At 7 and 14 days, the P2 group exhibited significantly lower values compared to	Different post-polymerisation procedures may affect the in-vitro cytotoxicity of 3D-print resin.

Study ID	Study design	Objective	Material tested	Manufacturing method (3D printer)	Sample size / shape	Sterilization / disinfection method	Cell line and test type	Extraction medium and incubation period	Results	Conclusions
		polymerisation conditions					Manassas, VA, USA);<br>Direct contact, MTT assay		the P1 and the C+groups (*P* < 0.001). At each time point, the cell's survival was significantly reduced in the P2 samples compared to the P1 samples (*P* < 0.001). Overall, the P1 post-curing process showed high cyto-compatibility after 7 and 14 days, with a cell viability of 107.12%±17.47% and 106.74% ± 18.41%, respectively. In contrast, the P2 procedures reported significantly moderate cytotoxicity (*P* < 0.001), with a	3D-printed aligners, post-cured using a Tera Harz Cure incorporating a nitrogen generator, were found to be biocompatible. In contrast, aligners post-cured using FormCure resulted in mild cytotoxicity

Study ID	Study design	Objective	Material tested	Manufacturing method (3D printer)	Sample size / shape	Sterilization / disinfection method	Cell line and test type	Extraction medium and incubation period	Results	Conclusions
									mean percentage of surviving cells of 59.79% ± 10.06% after 7 days, and 47.09% ± 20.62% after 14 days	
Kim et al. (2024)	In vitro	To investigated the effects of temperature and centrifugation time on the efficacy of removing uncured resin from 3D-printed clear aligners	Photo-polymerizable polyurethane resin (Tera Harz TC-85, Graphy Inc., Seoul, South Korea)	LCD 3D printer (UNIZ NBEE, UNIZ Technology LLC, USA)	n=5, aligners were cut into smaller pieces	EO gas	L929 (ATCC, CCL-1, American type culture collection, San Diego, CA, USA); Extract test, MTT assay	RPMI 1640 culture medium, 24h	The cell viability values are as follows: the positive control at 8.35 ± 0.20%, the negative control at 98.99 ± 8.78%, the NT group at 82.10 ± 4.46%, and the IPA group at 92.98 ± 1.64%. For the RT-2, RT-4, and RT-6 groups, the values are 89.31 ± 3.61%, 87.90 ± 6.80%, and 97.26 ± 2.06%, respectively. Similarly, the	Neither the temperature nor the duration of centrifugation cleaning significantly affected the optical properties, cell viability, or stress relaxation properties of the aligners. Based on these findings, it is recommended that centrifugal cleaning at 55 °C for 2 min with a force of

Study ID	Study design	Objective	Material tested	Manufacturing method (3D printer)	Sample size / shape	Sterilization / disinfection method	Cell line and test type	Extraction medium and incubation period	Results	Conclusions
									HT-2, HT-4, and HT-6 groups have cell viability values of 84.88 ± 9.04%, 91.02 ± 8.90%, and 87.61 ± 2.77%, respectively. Notably, the RT-6 and HT-4 groups exhibited significantly higher cell viability compared to the other experimental groups (P < 0.05)	27.95 g effectively removes uncured resin from aligners while maintaining clinically desirable esthetics
Migliorati et al. (2024)	Clinical trial (prospective observational pilot study)	This pilot study aimed to investigate the accuracy of teeth movement achieved with direct printed aligners	Tera Harz TC-85DAC resin (Graphy, Seoul, South Korea)	Phrozen Sonic XL 4k 2022 3D printer (Phrozen Technology, Hsinchu, Taiwan)	17 patients (8 males and 9 females) with a mean age of 27.67	N.A.	N.A.	N.A.	The overall accuracies for torque, tip, and rotation were 67.6%, 64.2%, and 72.0%, respectively. The accuracy of the change in transverse	The aligners were successfully planned and printed in-office and proved to be effective in treating mildly

Study ID	Study design	Objective	Material tested	Manufacturing method (3D printer)	Sample size / shape	Sterilization / disinfection method	Cell line and test type	Extraction medium and incubation period	Results	Conclusions
									diameter was 99.6%	misaligned cases. Direct printing of aligners represents a promising tool to enhance orthodontic treatment efficiency
Iodice et al. (2024)	In vitro	The aim of this in vitro study was to examine the potential impact of different curing times of 3D-printed orthodontic aligners on their cytotoxicity	Tera Harz TC-85 DAC resin (Grapy, Seoul, South Korea)	N.I.	n=60, square samples	Supernatant was filtered using a 0.22 mm pore size filter	Human gingival fibroblasts (HGF-1)-CRL2014 (ATCC, Washington, DC, NW, USA); Direct contac - immunoflorescence test after 3 days: alpha-tubulin antibody was applied for labelling to visualize overall cell shape. Additionally, a common blue fluorescent DNA dye (Hoechst) was used to facilitate nuclei iden- tification.	Human saliva, 14 days	Compared with the glass, only the 50-min curing time markedly reduced fibroblast cell growth. Additionally, a negative linear trend was observed between curing time and fibroblast growth. In comparison with the aligner control group, all samples, including the aligner control	3D directly printed aligners showed a cytotoxic effect similar to that of thermoformed conventional aligners in terms of fibroblasts growth. A linear trend was found between curing time and cells growth, indicating that directly printed align-

Study ID	Study design	Objective	Material tested	Manufacturing method (3D printer)	Sample size / shape	Sterilization / disinfection method	Cell line and test type	Extraction medium and incubation period	Results	Conclusions
							Extract test - MTT assay		samples, exhibited a significant reduction in the viability of human fibroblasts when exposed to saliva	ers could exhibit higher cytotoxicity if exposed to a longer curing time
Pasaoglu Bozkurt et al. (2024)	In vitro	This study aimed to compare and evaluate time-dependent biofilm formation and microbial adhesion on 6 different clear aligner systems	Tera Harz TC-85 (Graphy Inc, Seoul, South Korea)	N.A.	N.I.	N.I.	N.A.	N.A.	It was found that more bacterial formation occurred on ClearCorrect than on Smartee at 120, 168, and 240 hours (P<0.05). It was observed more biofilm formation at 168 hours on Graphy than on Smartee (P<0.05). It was found that S mutans 1 L acidophilus formed more biofilm at 120 and 168 hours	Elevated biofilm formation across all materials carries substantial clinical implications. Orthodontists and patients should remain aware of the increased risk of microbial colonization with extended aligner usage

Study ID	Study design	Objective	Material tested	Manufacturing method (3D printer)	Sample size / shape	Sterilization / disinfection method	Cell line and test type	Extraction medium and incubation period	Results	Conclusions
									on Graphy than on Invisalign (P<0.05)	
Wu et al. (2024)	In vitro	This study aimed to use a carboxybetaine methacrylate (CBMA) copolymer solution to surface treat 3D printed clear aligners at different fabrication stages, to impart antifouling properties, and assess the surface treatment at various fabrication stages' impact on physico-mechanical characteristics	Tera Harz TC-85 DAC (Graphy, Seoul, South Korea)	Uniz NBEE printer (Uniz, CA, USA)	n=5 (tensile test and micro vickers hardness test); n=5 (optical properties); n=8 (water contact angles); n=3 (confocal laser scanning microscope for salivary biofilm formation)	N.A.	Human gingival fibroblast-1 cell lines for indirect method, followed by MTT test after 24h; NCTC clone 929 (L-929) cells for direct contact	Cell culture medium (HyClone Co., UT, USA), 7 days	Surface treatment during CB1 stage exerted the most significantly unfavorable influence on properties of the 3D printed aligner resin. CB2 samples showed the maximum preservation of translucency even after 7-day aging. CB2 and CB3 phases showed enhanced hydrophilicity of sample surfaces with reduced adhesion of multispecies biofilm and S. mutans	Application of CCS surface treatment immediately after post-curing (CB2) can enhance the biofilm resistance of 3D printed clear aligners while maintaining high fidelity to optical translucency and constituent mechanical properties

Study ID	Study design	Objective	Material tested	Manufacturing method (3D printer)	Sample size / shape	Sterilization / disinfection method	Cell line and test type	Extraction medium and incubation period	Results	Conclusions
Knode et al. (2025)	Clinical trial (retrospective evaluation)	To evaluate the clinical effectiveness and efficiency of a directly printed aligner to correct moderate malocclusions and to evaluate the occurrence and nature of any complications associated with its use over 12 months	Tera Harz TC-85 (Graphy Inc, Seoul, South Korea)	Nbee 3D printer (Uniz Technology LLC, San Diego, Calif)	54 patients	N.A.	N.A.	N.A.	The mean number of aligners in the initial plan was 7.1 ± 2.9 and 5.1 ± 4.3 in maxillary and mandibular arches. Refinement was required in 40.8% (n = 20). The pretreatment PAR score of 17.01 ± 7.93 showed a significant improvement (86.6%), with a final PAR score of 2.25 ± 1.15. Minor complications were noted in 3 participants. The need for refinement was unrelated to the total number of aligners (odds	On the basis of this preliminary retrospective evaluation involving a single, experienced operator, DPAs may have a role in managing moderate malocclusions based on the PAR score reduction obtained. The need for refinement was unrelated to the total number of aligners used, whereas there was a weak negative association between the final PAR score and the

Study ID	Study design	Objective	Material tested	Manufacturing method (3D printer)	Sample size / shape	Sterilization / disinfection method	Cell line and test type	Extraction medium and incubation period	Results	Conclusions
									ratio, 1.05; 95% confidence interval, 0.94-1.18; $P = 0.36$). There was weak evidence of an association between the final PAR score and the total number of aligners (odds ratio, −0.03; 95% confidence interval, −0.07 to 0.003, $P = 0.07$)	number of aligners
Bleilöb et al. (2025)	In vitro	To evaluate whether design modifications that increase layer thickness require a longer UV curing time to ensure biocompatibility	Tera Harz TA-28 (Graphy Inc., Seoul, South Korea)	Asiga MAX 3D printer (Asiga SPS TM technology, Sydney, Australia)	n=36, circular specimens with a diameter of 1 cm and varying thicknesses of 0.5, 1, 2, 4 and 6 mm	N.I.	Human gingival fibroblasts; Extract test (12-day extracts from medium and saliva), AlamarBlue cell viability assay	Medium and saliva	Cell viability decreased with increasing specimen thickness (significant for 2 mm [$p < 0.001$], 4 mm [$p < 0.0001$], and 6 mm [$p < 0.01$]) under the manufacturer-recommended	The standard 20-minute UV curing protocol ensures the biocompatibility and patient safety of Tera Harz TA-28 for material thicknesses up to 6 mm

Study ID	Study design	Objective	Material tested	Manufacturing method (3D printer)	Sample size / shape	Sterilization / disinfection method	Cell line and test type	Extraction medium and incubation period	Results	Conclusions
									20-min UV curing. Extending the curing time did not improve cell viability. However, cell viability never decreased by more than 30%, meeting EN ISO 10993-5 standards for non-cytotoxicity	
Bor et al. (2025)	In vitro	This study aimed to evaluate the cytotoxicity of two resin materials, Tera Harz TC-85 DAC and Clear-A, along with the effects of two different post-printing protocols applied to Clear-A	Tera Harz TC-85 DAC (Graphy, Seoul, South Korea) and Clear-A (Senertek, Izmir, Turkey)	Ackuretta SOL printer (Ack- uretta, Taipei, Taiwan)	N.I., rectangular aligner samples (10 mm × 12 mm; thicknes: 0.6 mm)	70% ethanol solution for 5 min and rinsed with sterile de-ionized water	Human gingival fibroblast cell line (PCS-201-018TM); Extract test, XTT assay and xCELLigence Real Time Cell Analysis	DMEM, 72h	According to the XTT assay, undiluted resin extracts exhibited approximately 75–80% cell viability at 24 h, while further dilutions resulted in a viability exceeding 90%. No significant differences in viability were observed	This study demonstrated that extracts from all tested 3D-printed resins exhibited biocompatibility with human gingival fibroblasts. These findings support their potential for further applications in the dental and

Study ID	Study design	Objective	Material tested	Manufacturing method (3D printer)	Sample size / shape	Sterilization / disinfection method	Cell line and test type	Extraction medium and incubation period	Results	Conclusions
									among the groups at any dilution at 48 and 72 h. The xCELLigence RTCA results aligned with the XTT findings, showing a transient decrease in cell viability within the first 24 h, followed by continued cell growth	biomedical fields

N.I. = no information
N.A. = not applicable
